# Comparative effects of selected non-caffeinated rehydration sports drinks on short-term performance following moderate dehydration

**DOI:** 10.1186/1550-2783-7-28

**Published:** 2010-08-22

**Authors:** Peter G Snell, Robert Ward, Chithan Kandaswami, Sidney J Stohs

**Affiliations:** 1 University of Texas Southwestern Medical School, Dallas, TX, USA; 2 Sports Science Network, Dallas, TX, USA; 3 Castle Hills, TX, USA; 4 Creighton University Health Sciences Center, Omaha, NE, USA

## Abstract

**Background:**

The effect of moderate dehydration and consequent fluid replenishment on short-duration maximal treadmill performance was studied in eight healthy, fit (VO_2max _= 49.7 ± 8.7 mL kg^-1 ^min^-1^) males aged 28 ± 7.5 yrs.

**Methods:**

The study involved a within subject, blinded, crossover, placebo design. Initially, all subjects performed a baseline exercise test using an individualized treadmill protocol structured to induce exhaustion in 7 to 10 min. On each of the three subsequent testing days, the subjects exercised at 70-75% VO_2max _for 60 min at 29-33°C, resulting in a dehydration weight loss of 1.8-2.1% body weight. After 60 min of rest and recovery at 22 C, subjects performed the same treadmill test to voluntary exhaustion, which resulted in a small reduction in VO_2max _and a decline in treadmill performance by 3% relative to the baseline results. Following another 60 min rest and recovery, subjects ingested the same amount of fluid lost in the form of one of three lemon-flavored, randomly assigned commercial drinks, namely Crystal Light (placebo control), Gatorade^® ^and Rehydrate Electrolyte Replacement Drink, and then repeated the treadmill test to voluntary exhaustion.

**Results:**

VO_2max _returned to baseline levels with Rehydrate, while there was only a slight improvement with Gatorade and Crystal Light. There were no changes in heart rate or ventilation with all three different replacement drinks. Relative to the dehydrated state, a 6.5% decrease in treadmill performance time occurred with Crystal Light, while replenishment with Gatorade, which contains fructose, glucose, sodium and potassium, resulted in a 2.1% decrease. In contrast, treatment with Rehydrate, which comprises fructose, glucose polymer, calcium, magnesium, sodium, potassium, amino acids, thiols and vitamins, resulted in a 7.3% increase in treadmill time relative to that of the dehydrated state.

**Conclusions:**

The results indicate that constituents other than water, simple transportable monosaccharides and sodium are important for maximal exercise performance and effective recovery associated with endurance exercise-induced dehydration.

## Background

Both prolonged and intermittent exercise performed at high temperature increases metabolic rate and heat production [1], and culminates in dehydration [2]. The consequences of dehydration are the elevation of body temperature, steady increase in fluid and electrolyte losses, and the depletion of important nutrients, including muscle and hepatic glycogen [1-3]. Any fluid deficit that is incurred during one exercise session can potentially compromise the next exercise session if adequate fluid replacement does not occur. Therefore, it is exceedingly important to replace fluid and electrolyte losses, and replenish energy stores rapidly in order to achieve recovery before the advent of the next bout of exercise [3-5]. Fluid intake can attenuate or prevent many of the metabolic, cardiovascular, thermoregulatory and performance perturbations that accompany dehydration [6-8].

Ingestion of non-caffeinated sport drinks containing vital nutrients such as water, electrolytes and carbohydrate during exercise may help maintain physiological homeostasis [5,9-11], resulting in enhanced performance and/or reduced physiological stress on an athlete's cardiovascular, central nervous and muscular systems [8,11,12]. Both the volume of the rehydration fluid and its composition are critical in maintaining whole body fluid homeostasis. Ingestion of carbohydrates during prolonged exercise can aid performance, not only through increased glucose oxidation but also, indirectly, through enhanced water absorption [5]. Carbohydrates improve the rate of intestinal uptake of sodium, which in turn favors the retention of water [13]. When proper hydration status is maintained, the inclusion of carbohydrates in an oral rehydration solution delays the onset of fatigue during a subsequent bout of intense exercise in a warm environment [11,14].

Even modest (up to 2% of body weight) exercise-induced dehydration hampers aerobic performance capacity [11] and compromises cognitive capabilities [15,16]. The factors responsible for these effects may include plasma volume depletion leading to reduced venous pressure, reduced filling of the heart, elevation of core temperature, and depletion of electrolytes such as sodium, and possibly potassium. Information is scarce on the impact of rehydration on short-term work following dehydration. Armstrong et al. [7] assessed the effect of moderate (1.9 to 2.1% of body weight) dehydration induced by the drug, furosemide, on race times and maximal graded exercise test lasting about 12 min. There was a significant reduction in maximal test time while no changes were observed in maximal values for maximum oxygen consumption (VO_2max)_, heart rate (HR), ventilation (V) or lactate levels. Yoshida et al. [17] demonstrated that a critical water deficit threshold of 1.3 to 2.4% induced a significant decrease in aerobic fitness and maximal anaerobic power, which is dependent on non-oxidative pathways of adenosine triphosphate (ATP) production.

Nielsen et al. [18] studied physical work capacity after dehydration and hyperthermia, and concluded that the effects of elevated temperature, body water loss and prior exercise cannot easily be characteristically distinguished experimentally. These observations prompted us to design a protocol in which the temperature elevation of subjects during dehydration was allowed to recover, and which minimized prior exercise effects. The normal and dehydrated conditions were then compared using combined measures of performance and physiological responses.

We were interested in knowing the extent to which rehydration blunted performance perturbations following exercise and temperature-induced dehydration, when core temperatures were not elevated. A second aim of the study was to test our premise that certain amino acids, carbohydrate polymers, protective thiols and vitamins may evoke a performance advantage. Based on exercise capacity, we assessed and compared the effects of rehydration with commercially available non-caffeinated lemon flavored sports drinks, namely, Gatorade and Rehydrate Electrolyte Replacement Drink (AdvoCare International), using lemon flavored Crystal Light as the control rehydration fluid. These fluids vary in energy, electrolyte and nutrient content. The study was conducted using a blinded, placebo protocol.

## Methods

### Subjects

Eight healthy men, who participated regularly in competitive sports and were familiar with maximal treadmill testing, were recruited for this study. They were fully acquainted with the procedures of the study including risks and benefits before giving their consent. The research protocol was approved by the University of Texas Southwestern Medical Center Institutional Review Board. Their physical characteristics are depicted in Table 1.

**Table 1:** Subject characteristics at baseline visit

Subject	Age (yrs)	Ht (cm)	Wt (kg)	**VO**_**2max**_ **(mL.min**^**-1**^**)**	Maximal RER	Maximal **Heart rate (beats.min**^**-1**^**)**	**Maximal V**_**E **_**(L.min**^**-1**^**)**
1	22	193.0	81.6	3772	1.20	196	164.2
2	23	185.4	89.8	4347	1.21	208	158.6
3	28	182.9	79.4	3463	1.34	192	131.6
4	28	188.0	74.5	3049	1.27	175	130.5
5	39	182.9	96.1	4507	1.19	166	143.9
6	24	172.7	83.9	3236	1.23	NA*	105.8
7	23	175.3	84.4	3798	1.18	195	125.5
8	41	177.8	71.7	4531	1.07	170	139.5

Mean	28.5	182.4	82.7	3838	1.21	186.0	137.5
St Dev	7.5	6.8	7.9	575	0.08	15.7	18.7

### Experimental Design

A double blind placebo randomized within study design was used in this investigation. The experimental design involved an initial dehydration exercise bout of 60 min in hot conditions (27-33°C), followed by 60 min of recovery at about 22°C, prior to performing an individualized treadmill exercise test designed to induce exhaustion in 7-10 min. After the exercise test, the subjects were assigned 60 min to fully replace fluid losses (on a weight basis) from the previous exercise and then the same maximal exercise protocol was repeated. Gas exchange measurements were made using a metabolic cart (Medical Graphics, St. Paul, MN USA) during the exercise test to assess maximal oxygen consumption (VO_2max_), ventilation (V_E_) and respiratory exchange ratio (RER). In addition, heart rates (HR) were obtained at one min and three min intervals during the exercise and the recovery phases.

The study involved four visits to the laboratory, initially for measurement of maximal oxygen consumption (VO_2max_), and then to undertake a dehydration and rehydration protocol to measure the efficacy of the three rehydration conditions on performance. The protocol was as follows: 1) 60 min of moderate exercise in hot conditions (27-33°C); 2) 60 min of recovery, individualized maximum treadmill test to voluntary exhaustion; and 3) 60 min of recovery and rehydration with fluid (replacement of lost weight), followed by individualized maximum treadmill test to voluntary exhaustion.

During the first visit to the laboratory, the procedures were outlined and a 5 min treadmill warm-up was conducted to establish the treadmill speed that would be used for the graded maximal exercise test. This running pace corresponded to a maximal steady state effort, a heart rate (HR) of 150 beats per min (approximately 80% predicted maximal HR) and/or a perceived exertion of 15 on the Borg scale. After a 5 to 10 min rest, the subjects ran at their individualized pace starting at 0% grade, which was increased 2% every two min until voluntary exhaustion. Subjects were then assigned in random order to the three rehydration conditions. The investigator running the tests (PGS) was blinded to the rehydration conditions, as were the subjects. The composition of the sports drinks was similar in osmolality but varied per unit volume in terms of energy content, energy composition, electrolytes, vitamins and amino acids as shown in Table 2. The exact weight of fluid lost between the initial weigh-in and after the dehydration test was provided to the subjects who consumed the liquid in unmarked containers over approximately 30 min.

**Table 2:** Composition of Gatorade, Rehydrate and Crystal Light

Ingredient	Gatorade (240 mL)	Rehydrate (240 mL)	Crystal Light (240 mL)
Calories	50	49	5
Osmolality (mOsm)	290-303	274	NA
Total Carbohydrate (g)	14	12.5	0
Sugars (g)	14	9.7	0
Potassium (mg)	30	104	0
Sodium (mg)	110	104	35
Calcium (mg)	0	104	0
Magnesium (mg)	0	28	0
Chromium (as polynicotinate) (mcg)	0	5	0
L-Glutamine (mg)	0	209	0
Glutathione (mg)	0	50	0
L-Arginine (mg)	0	93	0
Pyridoxine alpha- ketoglutarate (mg)	0	105	0
Ubiquinone (coenzyme Q10) (mcg)	0	11	0
Thiamine (B1 - mcg)	0	160	0
Riboflavin (B2 - mcg)	0	178	0
Niacin (mg)	0	2	0
Pantothenic acid (B5 - mg)	0	1	0
Vitamin C (mg)	0	125	0
Vitamin A (as beta-carotene & vitamin A palmitate - IU)	0	1044	0
Other ingredients:	Sucrose syrup, fructose syrup, glucose, citric acid	Fructose, maltodextrin (2.8 g), malic acid, dextrose, sucralose, malic acid	

During subsequent visits to the laboratory, the subjects' weights were recorded without clothing. Subsequently, the subjects exercised for 60 min by either running outdoors in hot conditions, or indoors, alternately running for 10 min on a treadmill, and then riding a stationary Airdyne Cycle Ergometer for 10 min at a room temperature of 28°C to achieve a dehydrated and fatigued condition with an accompanying weight loss of 1.4 - 1.8 kg. During the third visit, two subjects, (JG and ZP), exercised indoors at 28°C alternating 10 min on a treadmill and Airdyne Cycle Ergometer. The remaining subjects easily ran 7.5 km outdoors in sunny conditions at about 32°C.

### Statistical Analysis

Standard statistical methods were employed for the calculation of means and standard deviations (SD). Descriptive data are presented as means ± standard deviation. Primary outcome measures (VO_2max _and treadmill time) were analyzed using repeated measures ANOVA of the difference between dehydration and rehydration values as the dependent variable. In addition, differences between the three drink replacements were compared using least square means from these models and adjusted for multiple comparisons with the Bonferroni correction to avoid type I error. The possible influence of dehydration level was tested with analysis of covariance. Significance in this study was set at P < 0.05.

## Results

The mean water loss during the initial dehydration phase ranged from 1.54 - 1.81 kg, corresponding to 1.8 - 2.1% loss in body weight (Table 3). This level of dehydration resulted in minimal effects on maximal HR and V for all individuals. Furthermore, no significant differences were observed in HR or V following rehydration with Crystal Light (control), Gatorade or Rehydrate (AdvoCare International) relative to either baseline values or values derived following dehydration (Table 3).

**Table 3:** Peak values during the treadmill performance test for heart rate* and ventilation at baseline, after dehydration and following rehydration

		**Heart Rate (beats.min**^**-1**^**)**			**Ventilation (L.min**^**-1**^**-btps)**		
Rehydrate	Wt loss (kg)	Baseline	Dehydration	Rehydration	Baseline	Dehydration	Rehydration
Mean ± SD	1.69 ± 0.54	186.0 ± 15.7	183.5 ± 12.0	185.5 ± 12.5	137.5 ± 18.7	134.1 ± 15.4	139.3 ± 18.0
							
Gatorade							
Mean ± SD	1.54 ± 0.63	186.0 ± 15.7	187.0 ± 14.5	183.0 ± 14.8	137.5 ± 18.7	136.4 ± 18.8	136.3 ± 21.4
							
Crystal Light							
Mean ± SD	1.81 ± 0.59	186.0 ± 15.7	183.5 ± 14.8	180.1 ± 14.3	137.3 ± 18.6	134.0 ± 17.9	134.2 ± 17.4

* Maximal HR not available at baseline.

Values for maximal oxygen consumption (VO_2max_) are provided in Table 4 as both mL.kg^-1^.min^-1 ^and mL.min^-1^. Relative to the baseline values, dehydration produced small but non-significant decreases in these values. Rehydration with Crystal Light (control) failed to restore VO_2max _to baseline values. Rehydration with Gatorade returned VO_2max _to slightly below baseline values, while rehydration with Rehydrate resulted in a VO_2max _(mL.min^-1^) that was 2.9% above the rehydrated state, and above baseline (Table 4). Although the differences were not statistically significant, the data suggested that the most favorable recovery was produced when Rehydrate was used for rehydration as compared to Gatorade and Crystal Light.

**Table 4:** Mean values ± SD for VO_2max _at baseline, after dehydration and following rehydration

	**VO**_**2**_**max (mL.kg**^**-1**^**.min**^**-1**^**)**		**VO**_**2**_**max (mL.min**^**-1**^**)**	
Baseline	46.6 ± 7.4		3,837.0 ± 575.5	
	Dehydrated	Rehydrated	Dehydrated	Rehydrated
	
Rehydrate	46.4 ± 5.5	46.6 ± 6.0	3,750.8 ± 501.4	3,861.3 ± 574.3
Gatorade	46.4 ± 0.7	46.4 ± 6.3	3,773.7 ± 555.9	3,826.5 ± 600.4
Crystal Light	45.7 ± 5.2	45.1 ± 5.6	3,697.9 ± 365.9	3,738.9 ± 449.0

**Figure 1:**
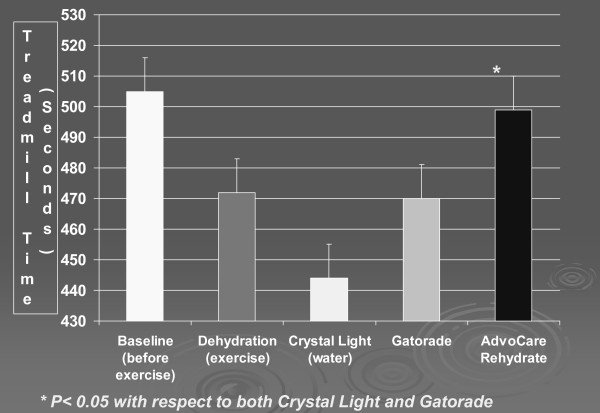
**Effects of rehydration with Crystal Light, Gatorade, and AdvoCare Rehydrate on treadmill performance as compared to baseline and dehydration performance**.

The effects of dehydration followed by rehydration with the three test beverages on treadmill times are presented in Figure 1. Dehydration resulted in an average 6.5% decrease in treadmill times relative to baseline. This decrease in treadmill time performance following dehydration was statistically significant (P < 0.002). Rehydration with Crystal Light resulted in a further 5.8% decrement in treadmill time performance. Rehydration with Gatorade resulted in a further decrease in treadmill time performance of 2.1% relative to the dehydrated state, which was 6.7% below baseline. Rehydration with Rehydrate resulted in a 7.3% increase in treadmill time relative to the dehydrated state, which was 1.1% below baseline (Figure 1).

**Figure 2:**
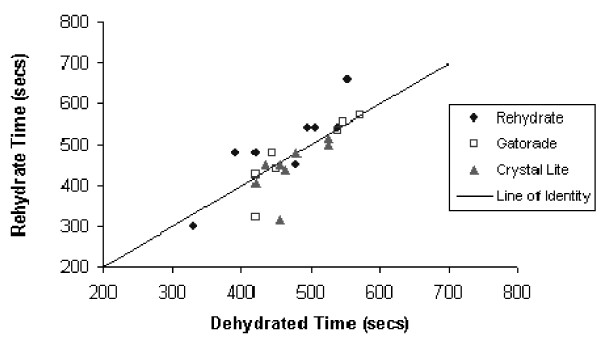
**Concordance plot showing dehydrated and rehydrated treadmill times for each subject**. Subjects above the line of identity improved with fluid replacement.

Evaluation of pair-wise differences for treadmill times following rehydration indicated that the differences between Rehydrate and both Crystal Light and Gatorade after adjustment for multiple comparisons (Bonferroni) were statistically significant (p < 0.001 and p < 0.016, respectively), while the difference in treadmill times between Crystal Light and Gatorade was not significant (p < 0.222). Figure 2 provides a concordance plot showing dehydrated and rehydrated treadmill times for each subject. Subjects above the line improved with fluid replacement, as was the case for the majority of individuals when their fluids were replaced with Rehydrate. The results suggest that composition of the rehydration fluid plays an important role in recovery and performance following moderate dehydration.

## Discussion

In the present investigation, we assessed the effects of prior endurance exercise-induced moderate dehydration and subsequent rehydration with two different ergogenic aids, Gatorade, which contains sodium, fructose and glucose, and Rehydrate, which contains fructose, glucose, maltodextrin, amino acids such as L-glutamine and L-arginine, various electrolytes and vitamins (qualitatively different carbohydrates and electrolytes), relative to a control fluid (Crystal Light containing sodium) on short-term performance (7 - 10 min) and energy expenditure. The order in which the three rehydration products were used was completely randomized, and as a consequence did not affect the results of the study. The results indicate that the effects of fatigue from the dehydration run and dehydration performance trial were not overcome by rehydration with Crystal Light, which is essentially a flavored water product, and in fact resulted in a decrease in performance.

It is unclear to what extent the differences in electrolytes in the three rehydration fluids (Table 2) contributed to the differences in performance (Figure 1). Crystal Light contains very little sodium and no potassium, calcium or magnesium. The Gatorade contains much less potassium and no magnesium or calcium relative to Rehydrate. The lack of sodium and potassium could have played a significant role in the decreased performance by Crystal Light. The osmolality of Gatorade and Rehydrate were similar, while Crystal Light was virtually devoid of an osmotic effect. These differences could have contributed to a resulting difference in the distribution of fluids both intracellularly and well as extracellularly, and subsequently influenced performance.

Rehydration with Gatorade produced an intermediate response in treadmill performance that was not significantly different from rehydration with Crystal Light. On the other hand, rehydration with Rehydrate was able to nullify the potential effects of fatigue from the dehydration run and improve treadmill time after limited dehydration, in comparison with that obtained from Gatorade and Crystal Light. Since there were no significant changes in peak HR, V or fluid volume, the observed performance enhancement upon rehydration with Rehydrate could not be accounted for by changes in these parameters. The results suggest that the quality, composition and content of the rehydration drink are crucial in modulating short-term endurance.

Few investigations designed to delineate the metabolic demands of short-term exercise exist due to methodological difficulties inherent in the establishment of steady state conditions associated with this type of exercise. The design of the present study combined a dehydration effect and a residual fatigue effect in order to provide conditions in which fluid, electrolyte and fuel replacement could confer beneficial effects. The decrease in treadmill time resulting from Crystal Light rehydration could be interpreted as residual fatigue since there were no differences in rehydration volumes among the three trials. The data indicate a moderate reduction in performance in dehydrated subjects (Figure 1).

The physiological parameter VO_2max_, a measure of aerobic capacity (the fastest rate at which the body utilizes O_2 _during heavy exercise) [19-21], is reduced only to a limited extent with the level of dehydration achieved in this study (Table 4). This moderate deficit in VO_2max _might signal the advent of fatigue as fatigue is often preceded by a plateau or even a decline in VO_2max _in the initial stages of the exercise task [22]. The change observed in VO_2max _following dehydration in the present investigation is consistent with that obtained by Buskirk et al. [23] and Saltin [24], although Craig and Cumming [25] documented a 10% reduction in VO_2max _with a similar degree of dehydration (1.9%). Enhanced physical fitness may be a factor in conferring additional protection against dehydration-induced decrements in VO_2max _because of the higher plasma volume in certain individuals who are physically more competent than others.

While rehydration with either Gatorade or Crystal Light resulted in values of VO_2max _lower than those of the baseline values, a moderate increase in VO_2max _occurred upon rehydration with Rehydrate. In athletic competition, the difference between a good performance and the best performance may be relatively narrow. Maughan et al. [26] concluded that performance improvements, although they may be minute, are critically important to the outcome of a race, and the athletes involved. For example, a good time for the mile run of 4 min 10 sec (250 sec) is only 4% slower than an elite-level time of 4 min. VO_2max _is a sensitive predictor of performance only when correlations are made among a broad range of abilities. Furthermore, a comparison of the VO_2max _of top runners revealed no relationship between VO_2max _and race times [27].

The provision of glucose polymers (maltodextrin) as transportable carbohydrates in addition to fructose in Rehydrate might have conferred some performance benefits. The generally higher gastric emptying rate of glucose polymer solutions than that of free glucose solutions [28] may result in increased intestinal absorption and nutrient supply to the active muscles [10]. Solutions containing glucose polymers possess a higher energy density than simple sugar containing beverages with similar osmolality [29] and also show the ability to maximize glycogen re-synthesis in the muscles [10]. Glucose polymers undergo degradation to glucose by salivary and pancreatic amylases and mucosal glucoamylase in the upper gastrointestinal tract, resulting in a more prolonged absorption, utilization and oxidation than that obtained with simple sugars [30,31]. The rate of oxidation of maltodextrin is higher than that of fructose [10,32]. Their combination, however, may facilitate sustained conversion/oxidation in the body and produce higher oxidation than that obtained with single carbohydrates [33], delaying the onset of fatigue, sparing endogenous carbohydrate reserves, and thus enhancing endurance.

Both oral L-glutamine and oral glucose polymer, present in Rehydrate, promote the storage of muscle glycogen while the ingestion of L-glutamine and glucose polymer together enhance the storage of carbohydrate outside of skeletal muscle [34,35], the most feasible site being the liver. The metabolism of L-glutamine is an indicator of pyruvate generation and metabolic capacity during cycling exercise in humans [36]. The reduction of plasma L-glutamine, an anaplerotic substrate, seems to be a harbinger of severe exercise-associated stress. Its availability modulates glucose homeostasis during and after exercise and thus could have implications for post-exercise recovery [37]. Some of the effects of L-glutamine may be mediated through the cytokine, IL-6, an immunoregulatory polypeptide implicated in the maintenance of glucose homeostasis, muscle function and muscle cell preservation during intense exercise. Plasma levels of L-glutamine decline during exercise, which in turn can decrease IL-6 synthesis and release from skeletal muscle cells. L-Glutamine administration during the exercise and recovery phases prevents the depression in L-glutamine, and consequently enhances the elaboration of IL-6 [38].

Both AMP-activated protein kinase (AMPK) and IL-6 appear to be independent sensors of a low muscle glycogen concentration during exercise [39]. AMPK is a key metabolic sensor in mammalian stress response systems and is activated by exercise [40]. IL-6 activates muscle and adipose tissue AMPK activity in response to exercise [39,41]. AMPK activation could lead to enhanced production of ATP via increased import of free fatty acids into mitochondria and subsequent oxidation [42]. These observations indicate the potential benefits of L-glutamine in up-regulating cellular IL-6 production and activating AMPK, which modulates carbohydrate uptake and energy homeostasis.

Yaspelkis and Ivy [43] reported that L-arginine supplementation could enhance post-exercise muscle glycogen synthesis and exert potential positive effects on skeletal muscle recovery after exercise, possibly by augmenting insulin secretion and/or carbohydrate metabolism. Accruing evidence attests to the role of endothelial nitric oxide (NO), produced from L-arginine, in energy metabolism and augmenting performance [44]. The central blockage of NO increases metabolic cost during exercise, diminishes mechanical efficiency and attenuates running performance in rats [45]. Other investigations [46] document that AMPK-induced skeletal muscle glucose uptake is dependent on NO, indicating the potential positive effects of L-arginine in muscle metabolism and function, with implications for endurance. Provision of L-arginine during rehydration with Rehydrate might be beneficial in maintaining cardiac and skeletal muscle blood flow [47]. These pharmacological actions might mitigate the potential impact of impending fatigue during a maximal exercise task. The coordinated function of some of the metabolically connected nutrients included in Rehydrate may be pivotal not only for cellular energy transduction but also for muscle cell preservation and the maintenance of cellular homeostasis.

## Conclusions

In summary, information garnered from this study suggests that a rehydration medium comprising transportable monosaccharides, fructose and dextrose, glucose polymer (maltodextrin), the electrolytes sodium and potassium, conditionally essential amino acids and a host of other nutrients results in enhanced performance, which has implications for success in a competitive setting. The constituents of this drink, therefore, harbor the potential to blunt metabolic and physiological perturbations, and ameliorate performance decrements. The recognized pharmacological effects of some of the important nutrient constituents of this rehydration beverage might provide a basis for their presumed and purported roles in exercise performance.

## List of Abbreviations

VO_2max_: maximum oxygen consumption; HR: heart rate; V_E_: ventilation; RER: respiratory exchange rate; NO: nitric oxide; AMPK: AMP activated protein kinase;

## Competing interests

The authors declare that they have no competing interests.

## Authors' contributions

PGS made substantial contributions to the experimental design, data acquisition, interpretation of the data and drafting of the manuscript. RW made major contributions to the experimental design, data acquisition, and interpretation of the data. SJS contributed to the conception of the study, interpretation of the data, and drafting of the manuscript. CK was involved in the conception of the study, data interpretation, literature review, and drafting of the manuscript. All authors read and approved the final manuscript.
